# Relation Between Injection Molding Conditions, Fiber Length, and Mechanical Properties of Highly Reinforced Long Fiber Polypropylene: Part II Long-Term Creep Performance

**DOI:** 10.3390/polym17121630

**Published:** 2025-06-12

**Authors:** Jon Haitz Badiola, U. Astobitza, M. Iturrondobeitia, A. Burgoa, J. Ibarretxe, A. Arriaga

**Affiliations:** 1 Leartiker S.Coop, Basque Research and Technology Alliance (BRTA), 48270 Markina-Xemein, Spain; uastobitza@leartiker.com (U.A.); aburgoa@leartiker.com (A.B.); aarriaga@leartiker.com (A.A.); 2 LCTG, Life Cycle Thinking Group, University of the Basque Country (UPV/EHU), 48013 Bilbao, Spain; maider.iturrondobeitia@ehu.eus (M.I.); julen.ibarretxe@ehu.eus (J.I.); 3 Department of Graphic Design and Engineering Projects, University of the Basque Country (UPV/EHU), 48013 Bilbao, Spain; 4 Department of Applied Physics, University of the Basque Country (UPV/EHU), 48013 Bilbao, Spain

**Keywords:** long fiber thermoplastics, injection molding, microstructure, DMA, creep

## Abstract

This study investigates the long-term mechanical performance of highly reinforced long glass fiber thermoplastic polypropylene composites, focusing on the effects of processing parameters, fiber length, and skin–core structures. Dynamic mechanical and creep analyses were conducted to evaluate the impact of injection molding on the final microstructure and long-term mechanical properties. The findings confirm that a significant microstructural change occurs at a fiber length of 1000 µm, which strongly influences the material’s mechanical behavior. Samples with fiber lengths above this threshold reveal greater creep resistance due to the reduced flowability that leads to more entangled, three-dimensional fiber networks in the core. This structure limits chain mobility and consequently improves the resistance to long-term deformation under load. Conversely, fiber lengths below 1000 µm promote a planar arrangement of fibers, which enhances chain relaxation, fiber orientation, and creep strain. Specifically, samples with fiber lengths exceeding 1000 µm exhibited up to a 15% lower creep strain compared to shorter fiber samples. Additionally, a direct relationship is observed between the findings in the viscoelastic response and quasi-static tensile properties from previous studies. Finally, the impact of the microstructure is more pronounced at low temperatures and becomes nearly negligible at high temperatures, indicating that beyond the glass transition temperature, the microstructural effect diminishes gradually until it becomes almost non-existent.

## 1. Introduction

Polypropylene (PP) has emerged as one of the most widely used thermoplastic polymers in recent decades, owing to its excellent properties, cost-effectiveness, and ease of processing. Ongoing advancements in its formulation and processing techniques have significantly broadened its applications across diverse sectors, including the biomedical, construction, and automotive industries. This widespread use is largely attributed to PP’s unique combination of characteristics, such as outstanding chemical resistance, low density, and good fatigue performance [1]. In structural applications, PP is commonly employed as a matrix material reinforced with various fibers, including glass fiber (GF) and carbon fiber (CF). These fiber-reinforced PP composites are frequently utilized in the production of structural components such as pipes or automotive bumper beams, where enhanced mechanical performance is required [2,3].

Long fiber thermoplastics (LFTs) combine the fast production times of injection-molded parts with enhanced mechanical and thermal properties, approaching those of continuous fiber composites. This technology consists of feeding the injection molding (IM) machine with chopped pellets where their fiber length is the longest that can be processed via IM.

Fiber length plays a crucial role in determining the final properties of the composite, highlighting the importance of processing parameters during injection molding [4,5]. This fiber length has been found to directly influence the dominant failure mechanisms in LFT composites [6,7], relative to whether the resulting fiber length is longer than the critical fiber length or not, which results in either fiber breakage or fiber pullout, respectively [8]. Thermoplastic composites, having fibers shorter than 1 mm, are classified as short fiber reinforced thermoplastics (SFRPs). These composites are the most used materials in the injection molding industry. On the opposite end of the spectrum are long fiber composites, where the fiber length goes from 25 mm to continuous mats. These materials cannot be processed using injection molding and require alternative manufacturing techniques, such as compression molding.

**Figure 1:**
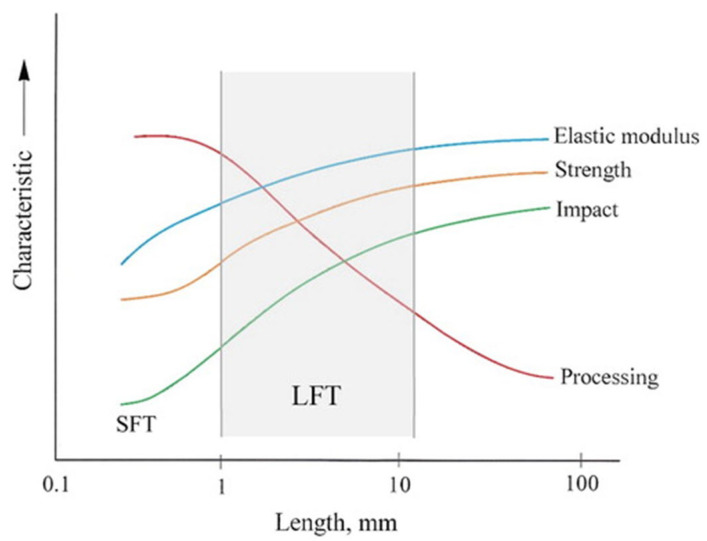
Relation between fiber length, mechanical properties, and processing for SFTs and LFTs.

Between these two extremes lies long fiber thermoplastics (LFTs), where the pellets are produced via pultrusion and then chopped to lengths between 8 and 25 mm. These can be processed using IM; however, processing parameters and mold designs are critical due to the significant fiber breakage during IM. Controlling fiber breakage during IM is essential to preserve the mechanical advantages of these composites [9]. Figure 1 points out the relationship between fiber length, ease of processing, and mechanical performance in short and long fiber thermoplastics.

It is well known that the fountain flow effect on the composites creates skin–core structures during the filling stage of injection molding [10,11]. However, the filling processes for traditional SFTs and LFTs differ significantly, with a strong correlation between the fiber aspect ratio and the thickness of the skin and core regions [12]. These differences in the three-layered structure leads to variations in the final mechanical properties. Consequently, controlling the fiber length during the injection process is essential to regulate the skin-to-core ratios and establish their relationship with the resulting mechanical properties. Several studies have focused on establishing a correlation between the three key factors of LFT composites: injection parameters, microstructure, and mechanical properties [13,14,15,16,17,18,19,20]. The skin–core structure directly influences the fiber orientation distribution [16]. Following this line of investigation, almost no studies were found that try to correlate these three factors with viscoelastic or long-term performance properties, such as dynamical mechanical analysis (DMA) or the Time Temperature Superposition Principle (TTSP) for creep testing.

On the one hand, dynamic mechanical analysis (DMA) is a widely used technique for studying the viscoelastic behavior of materials, and several studies have extensively investigated the dynamic behavior of various LFT polymer composites [21,22,23]. These studies were carried out using the same injection parameters, and, therefore, the same skin–core relationships were studied. To gain a deeper understanding of the dynamic behavior of LFT composites, specific dynamic tests were conducted in this study.

On the other hand, long-term mechanical analysis is essential for predicting a product’s lifespan, encompassing evaluations such as fatigue, creep, or thermal stability. The long- term mechanical performance of SFRP composites has been widely studied [24]. Numerous studies have investigated the fatigue failure mechanisms of short fiber-reinforced polymers (SFRPs) and their corresponding modeling approaches [6,25,26]. Conversely, similar studies on LFT composites are much scarcer [8,20]. The thermal stability of PPGF composites has been widely studied, concluding that interfacial adhesion, fiber length, and fiber content are the main factors [27,28,29]. Most studies regarding creep behavior are focused on SFRP composites [30,31,32,33] or the compression of LFT composites with fibers longer than 10 mm [34,35,36,37]. It is widely accepted that LFT composites exhibit better impact resistance behavior than SFRP composites [38,39,40,41,42,43], but no research has been found correlating long-term mechanical properties with the injection parameter-dependent microstructure of glass-fiber-reinforced LFT polypropylene (PP) composites.

Considering the strong relationship between injection parameters, microstructure, and mechanical properties in fiber-reinforced composites [16] and the lack of literature addressing long-term properties in this area, the aim of this work was to study the long-term performance of LFT composites in relation to processing parameters, the resulting fiber length, and skin–core structures.

## 2. Materials and Methods

### 2.1. Materials

The material selected for this work was a STAMAX 50YM240, supplied by Sabic (Sabic JSC, Riyadh, Saudi Arabia), which is a 50 wt % glass fiber-reinforced homopolymer PP. The pellets are 10 mm-long LFTs, obtained via pultrusion. The final composite Melt Flow Index is 3.8 g/10 min under a 5 kg load and at 230 °C. Table 1 presents selected material properties as provided in the manufacturer’s data sheet.

### 2.2. Processing

The injected workpiece was a 300 × 100 × 3.2 mm^3^ plate, from which samples were milled from the end section to maximize fiber orientation. The samples were injected using an Engel Insert 200 (Engel Austria GmbH, Schhwertberg, Austria) injection molding machine equipped with a 55 mm-diameter standard design screw.

According to our previous work [16], the greatest influence on fiber breakage occurs during the dosing stage, with minimal impact from the injection speed. Table 2 summarizes the injection molding parameters studied (a 2^2^ factorial design). The selection of backpressure (P_back_) and screw tangential speed (V_f_) as the primary variables was based on earlier findings [16], which indicate that these parameters have the most significant influence on fiber breakage during the dosing stage.

### 2.3. Specimen Preparation

**Figure 2:**
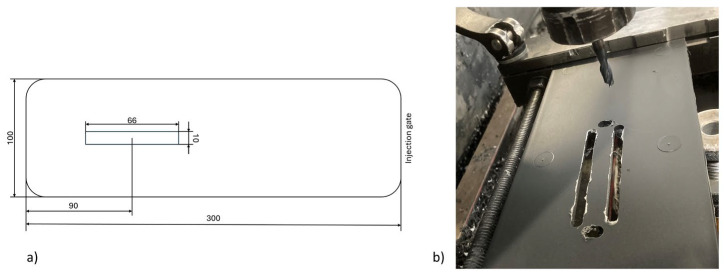
(**a**) Schematic representation of the sample machining plan. (**b**) Machining process of the samples.

A Fidia Cortini HS 664 (Fidia S.p.A., San Mauro Torinese, Italy) milling machine was used to cut dog-bone shaped ISO 8256 [44] type 3 (tensile) and rectangular ISO 179-1 [45] (impact) specimens from the injected plates. The center of the specimens was located at 90 mm from the filling end for both types of samples, as shown in Figure 2. The same position was used for all the samples to avoid any influence of fiber orientation resulting from the front flow advance.

### 2.4. Fiber Analysis

To carry out the fiber length measurement, the specimens (whole impact specimen) were pyrolyzed in a Nabertherm HT16-17 (Nabertherm GmbH, Lilienthal, Germany) furnace at 675 °C for 4 h. Afterward, the fibers were dispersed in a solution of water and glycerine. A representative sample size of more than one thousand fibers was measured and counted using a Nikon SMZ45T (Nikon Corporation, Nishioi, Shinagawa-ku, Tokyo, Japan) optical microscope and the NIS-Elements BR analysis software, 3.22.15 version.

The fiber length distribution, its weight average fiber length (l_w_), the number of average fiber lengths (l_n_), and the fiber length ratio (FLR) between l_w_ and ln were calculated. Each measurement included more than 1000 fibers to ensure a representative sample. The three parameters were obtained using the formulas provided below.

(1) $$l_{n}=\frac{\sum n_{i}l_{i}}{\sum l_{i}}$$

(2) $$l_{w}=\frac{\sum n_{i}l_{i}^{2}}{\sum n_{i}l_{i}}$$

(3) $$Fiber\;Length\;Ratio\;\left(FLR\right)=\frac{l_{w}}{l_{n}}$$

### 2.5. Micro Computed Tomography (MicroCT)

MicroCT was used to analyze the fiber orientation of the samples. Pieces of 10 × 10 × 3.2 mm were cut from the center of the impact test specimens and scanned in an RX Solutions EasyTom “XL” scanner (RX solutions NDT, Chavanod, France), with a voxel size of 5 µm. The tomograms were analyzed using the Fiber Composite Materials Module of VGStudio by Volume Graphics (Volume Graphics GmbH, Heidelberg, Germany), version 2024.1.

The orientations of the samples were defined as follows: the X-direction corresponds to the flow front direction, the Y-direction represents the transverse direction (perpendicular to the flow front), and the Z-direction is defined as the through-thickness direction of the samples.

### 2.6. Dynamic Mechanical Analysis (DMA)

DMA tests were conducted using a Metravib DMA +300 (Metravib, Acoem Group, Limonest, France) machine with specific test bars measuring 66 × 10 × 3.2 mm in tension mode. The storage modulus (E’), loss modulus (E’’), and loss tangent (t_g_[α]) as a function of temperature were measured at a 10 Hz oscillating frequency while heating from −70 °C to 150 °C at 10 °C/min. All experiments were performed at a dynamic amplitude of 10 µm to perform the tests within the linear viscoelastic region of the material. All experimental results presented are the mean values of five samples.

### 2.7. Creep Tests

Creep tests were also performed in a Metravib DMA +300 (Metravib, Acoem Group, Limonest, France) test instrument with 66 × 10 × 3.2 mm tensile bars. A 2 MPa static force was applied to each sample at different temperatures, ranging from room temperature (23 °C) to 140 °C in 10 °C steps. The stress level was applied for 15 min, followed by a recovery period of another 15 min, with strain recorded throughout the entire process. The individual strain vs. time curves obtained at each temperature were processed automatically by horizontal time shifting to obtain master curves spanning longer test durations, targeting a lifespan in the order of 30 years of stress exposure. The master curves were fitted using the two widely known models for time temperature superposition (TTSP): Williams, Landel and Ferry (WLF) and Arrhenius. As with the other tests, all experimental results presented are the mean values of five samples.

## 3. Results and Discussion

### 3.1. Fiber Length Analysis

According to Table 3, the fiber length analysis revealed four distinct fiber length distribution ratios in ascending order, ID 6, 8, 2, and 4. This range of ratios aligns with previous findings [16], indicating that variations in fiber length will result in differences in skin–core ratios across the thickness of the injected plates. The combination of a high backpressure and a low screw tangential speed represents the most detrimental conditions for fiber-reinforced composites, resulting in fiber lengths within the SFRP range. As shown in Table 3, the longest fiber lengths are achieved under low backpressure conditions. Furthermore, the combination of a low backpressure and a high tangential screw speed yields the most favorable scenario for preserving the fiber length during injection molding.

In injection molding, backpressure is essential for ensuring consistent dosing from cycle to cycle. However, identifying the minimum effective backpressure is critical to minimize fiber breakage during processing.

To ensure clarity and organization of the results, the samples will be identified and categorized based on their weight-average fiber length (l_w_). Throughout the discussion of the results, the samples will be referred to as follows: ID 2 will be designated as “l_w_ 1500”, ID 4 as “l_w_ 1700”, ID 6 as “l_w_ 900”, and ID 8 as “l_w_ 1300”.

### 3.2. Fiber Orientation Analysis

**Figure 3:**
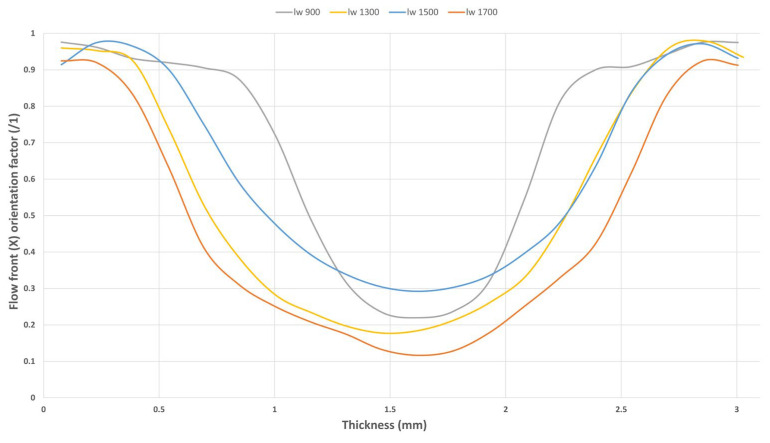
Evolution of the tensor component of filling direction (X) for each sample.

The results of the CT scan, shown in Figure 3, demonstrate a strong correlation between fiber length and the skin–core ratios in each sample. Samples with the shortest fiber length (l_w_ 900) exhibit the thickest oriented region (skin) and the thinnest perpendicularly oriented region (core). As the fiber length reaches approximately 1300 µm (l_w_ 1300), the skin–core regions change dramatically, resulting in thick core regions combined with thin skin regions. Beyond this threshold, samples with longer fiber lengths show no significant differences in microstructure.

This study illustrates that the transition from one microstructure to another occurs around the 1000 µm mark. However, further work may be necessary to determine whether this is an abrupt change at 1000 µm or if a transition phase exists between 800–1300 µm. Once the fiber length reaches this value, the material’s flowability changes, and the entanglements generated during flow alter the structures formed inside the cavity. These differences between skin and core regions between the samples are expected to have a direct impact on the dynamic and creep responses of the material.

#### 3.2.1. Fiber Entanglement

**Figure 4:**
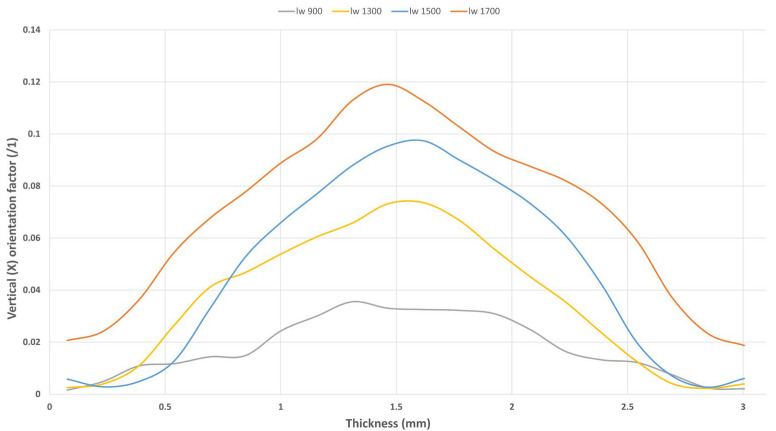
Measured through-thickness fiber orientation and evolution of the tensor component’s vertical direction to the filling plane (Z) for each sample.

Fiber entanglement can also be observed in the percentage of Z-oriented fibers (Figure 4). In samples with short fibers, such as “l_w_ 900”, almost no vertically oriented fibers are present across the thickness. However, as the fiber length increases to 1500 µm, the proportion of Z-oriented fibers rises to 10%, indicating that the number of Z-oriented fibers is three times higher in the long fiber microstructure. This suggests that the material’s flowability decreases with longer fibers, preventing them from orienting in a planar (2D) manner as the short fibers do. Consequently, this leads to more entanglement and thicker core regions.

The higher content of Z-oriented fibers, combined with thicker perpendicularly oriented core regions, directly affects the material’s mechanical behavior. These microstructural changes create a 3D network in the core region that enhances the material’s creep resistance, leading to lower strain levels. On the other hand, short fibers with high orientation capabilities but almost no Z-oriented fibers result in 2D planar microstructures, exhibiting higher dynamic responses.

#### 3.2.2. Skin/Core Relationship

Considering each sample is a three-layered structure, the boundary of the skin/core region was defined as the point where the X-oriented fibers exhibited an orientation factor value (X) below 0.8.

**Figure 5:**
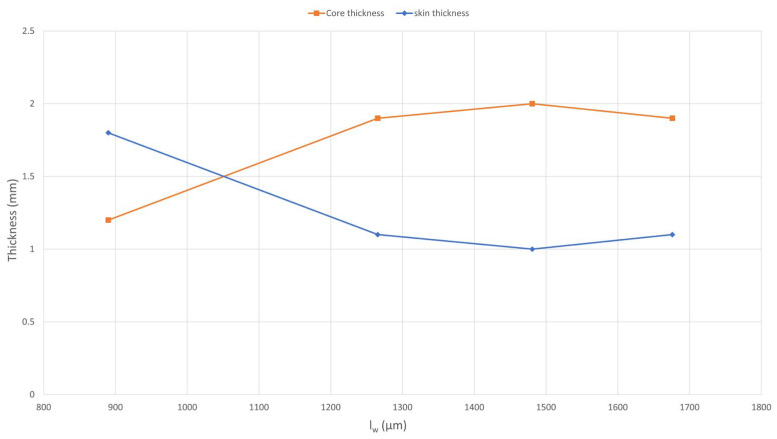
Cumulative thickness of skin and core regions on each sample.

The results reveal, in Figure 5, a significant structural variation at a critical fiber length (l_w_) of 1000 µm. For fiber lengths below this threshold, the injection molding process generates thicker, fully oriented skin regions. This phenomenon is attributed to the enhanced flowability of the material, which increases the orientation capacity of the fibers, thanks to the reduced average fiber length. Under these conditions, fibers align more effectively in the flow direction.

On the other hand, all subsequent samples exhibited thicker core regions. These longer fibers reduced the melt’s flowability and its capacity for orientation. Consequently, the resulting structures featured more fibers aligned along the *Z*-axis and thicker core regions.

### 3.3. Dynamic Mechanical Analysis (DMA)

#### 3.3.1. Storage Modulus (E’)

**Figure 6:**
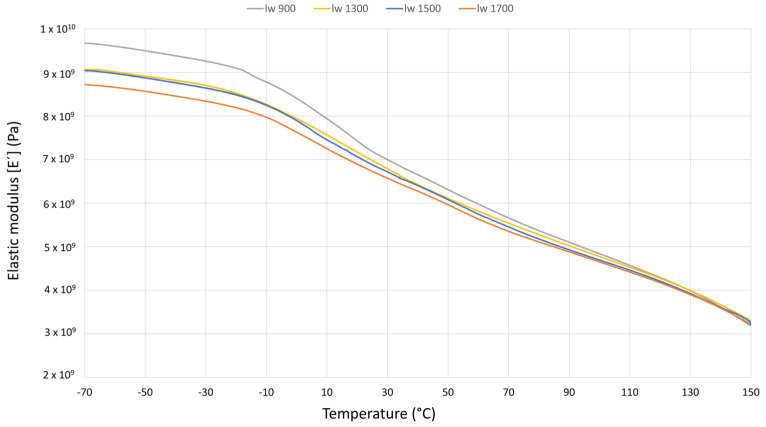
Dynamic results: comparison of the storage modulus in the temperature sweeps for each sample.

In Figure 6, the differences between the storage modulus of each sample can be observed. According to previous work [16], a direct relation between tensile properties at room temperature and storage modulus is evident. Thicker stress-oriented skin layers have a greater impact on storage properties than fiber length effects. The sample with the highest E’ was the one with the shortest fibers (l_w_ 900).

Examining the temperature profile, significant differences in storage modulus between the samples are observed at low temperatures. The highly oriented thick skin region of “l_w_ 900” contributes to the microstructure, yielding the highest storage modulus values. Additionally, a notable slope change is observed around the material’s glass transition temperature (0 °C). On the other hand, at high temperatures, the difference between the storage modulus of each sample decreases, becoming almost negligible at around 80 °C. These results show that skin–core effects are strongly dependent on temperature. At high temperatures, such as 80 °C and above, the skin–core effects become almost insignificant. Above this temperature, the fiber/matrix ratios become critical, showing almost no differences between the samples.

Therefore, the DMA findings indicate that although longer fibers generally enhance stiffness, in this case, the dynamic mechanical response at low temperatures is primarily governed by the skin–core effect [16]. Samples with shorter fibers (e.g., l_w_ 900) develop thicker, highly oriented skin layers, which contribute more significantly to the storage modulus (E’) than fiber length itself. This occurs because the skin region aligned with the flow direction bears most of the dynamic load. Thus, it can be concluded that at low temperatures, the skin–core structure is the dominant factor influencing E’, whereas at higher temperatures, its influence diminishes, and the fiber–matrix ratio becomes more significant.

#### 3.3.2. Loss Modulus (E’’)

**Figure 7:**
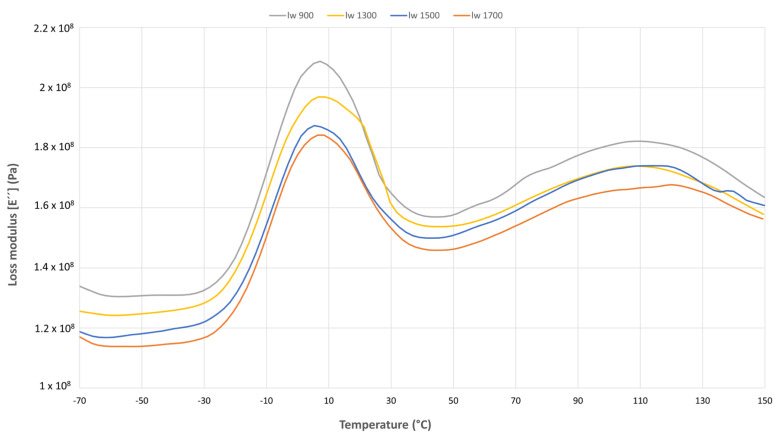
Loss modulus comparison between all the samples.

Following the results obtained in the storage modulus behavior of the material, the pattern observed in the loss modulus, Figure 7, remains the same. These results indicate that the damping properties of the material could also be affected by the skin–core regions. The shortest fibers (l_w_ 900) exhibit the highest loss modulus, with a decreasing trend until the longest fibers (l_w_ 1700). This reduction in the loss modulus can be related to the increment of the core regions and Z-oriented fibers, creating a 3D network structure that reduces the chain interaction between the layers. The reduced quantity of Z-oriented fibers observed in the “l_w_ 900” sample results in a planar (2D) microstructure with enhanced chain mobility across the layers throughout the thickness. This structural feature is associated with an increase in the sample’s loss modulus. This viscoelastic behavior may be related to the creep results obtained (Section 3.4).

#### 3.3.3. Loss Tangent (Tg[α])

**Figure 8:**
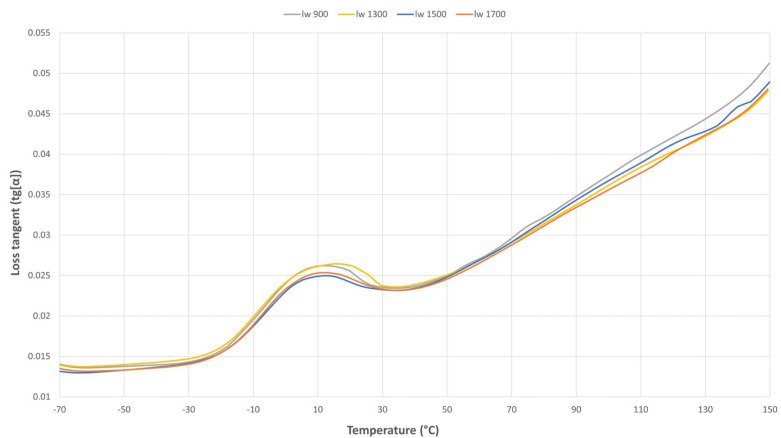
Loss tangent results of the four samples.

The loss tangent measurements showed in Figure 8, indicate that as a highly reinforced composite containing up to 50% glass fiber, variations in the loss tangent remain minimal up to 70 °C, with negligible differences observed among the samples over a broad temperature range. However, at higher temperatures, more pronounced variations in the loss tangent become evident. Notably, the “l_w_ 900” sample exhibits the highest loss tangent peak, suggesting enhanced chain mobility.

### 3.4. Creep Tests

**Figure 9:**
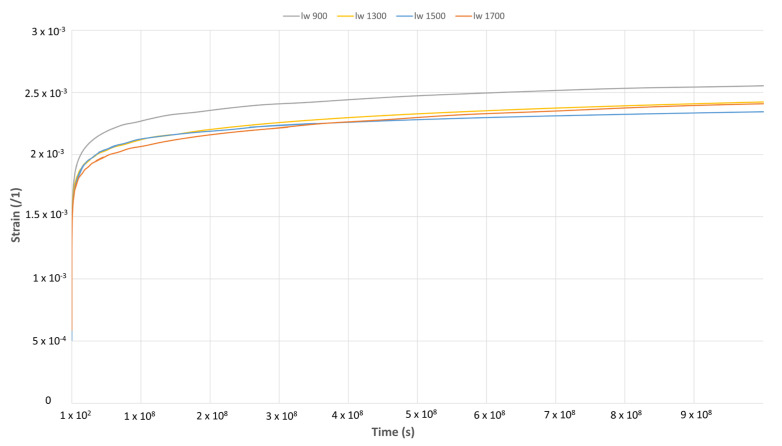
Comparison of the creep master curves for the different samples at 23 °C and at a stress level of 2 MPa.

The master curve presented in Figure 9 highlights key insights into the relationship between fiber length, microstructure, and creep behavior. The sample with the lowest fiber length, “l_w_ 900”, exhibits the highest creep strain, despite having the highest storage modulus (E′). This counterintuitive behavior is attributed to the reduced presence of Z-oriented fibers, which promotes a predominantly two-dimensional planar fiber orientation. In the absence of Z-oriented entanglements, the composite structure allows for greater molecular mobility and enhanced energy dissipation, as evidenced by the increase in loss modulus (E’’). Consequently, the material’s creep resistance is diminished, as the layered architecture facilitates interlayer movement and deformation under sustained loadings. This observation underscores the critical role of fiber orientation in governing the viscoelastic response of long fiber thermoplastics.

In contrast, the materials with fiber lengths over 1000 µm demonstrate improved creep resistance. This improvement is primarily due to thicker entangled cores that enable effectively reducing chain relaxation. Additionally, these samples exhibit lower energy dissipation, indicating more efficient energy transfer within the microstructure. A threshold effect is observed at l_w_ ≈ 1000 µm, where further increases in fiber length provide diminishing returns in enhancing creep resistance. This suggests that the transition in microstructure plays a pivotal role, and beyond a certain point, additional fiber length no longer yields proportional improvements in performance.

## 4. Conclusions

This study provides a deeper understanding of the micromechanical effects of injection molding and their influence on the final long-term mechanical properties of injection-molded LFTs. The results demonstrate that the skin–core ratios of the final product directly influence both viscoelastic and long-term mechanical properties. However, the influence of processing parameters should not be underestimated. The parameters used, particularly during the dosing stage of the injection process, significantly affect the final microstructure of the part and, consequently, its ultimate properties.

A low backpressure combined with a high tangential speed results in the lowest fiber breakage values. Long-term mechanical analysis indicates that fibers longer than 1000 µm are sufficient to achieve optimal long-term creep properties (lowest strain increase in creep testing). This suggests that at nearly any rotation speed used, the fiber lengths obtained are adequate, highlighting the critical role of backpressure in fiber damage and its subsequent impact on long-term properties. The ability to create these 3D entanglements during injection molding can increase the material’s creep resistance by up to 15% compared to traditional SFRP materials. Controlling fiber breakage during injection molding of LFT materials is crucial to preserving their distinct mechanical behavior compared to SFRP compounds.

The 2D planar arrangement of fibers with lengths below 1000 µm facilitates chain mobility between the layers of the sample. This behavior not only increases the loss modulus but also significantly reduces the material’s creep resistance. Over the long term, the presence of Z-oriented fibers plays a crucial role in maintaining the structural integrity of the material by enhancing its creep resistance. Despite a 10% increase in the elastic modulus of the materials with the shortest fibers, the key factor driving market interest in LFTs is the improvement in long-term properties, attributed to the threshold generated during injection in the core regions. Due to this, there is no point in obtaining LFT composites to break up the fibers and create mechanical responses like SFRPs.

The three-layered microstructure induces significant changes in the mechanical behavior of the material. The elastic modulus (E’) indicates that the microstructure-dependent mechanical behavior is more pronounced at temperatures below the glass transition temperature (T_g_). However, above T_g_, these differences diminish progressively and become negligible at temperatures around 70 °C. At these higher temperatures, the fiber-to-matrix ratio becomes the dominant factor influencing the material’s mechanical behavior, rather than the microstructure itself.

The application of LFT materials is closely linked to long-term mechanical performance requirements. Therefore, the proper injection molding of these materials is crucial to ensuring the correct functionality of the parts. Low backpressures combined with high tangential speeds help reduce the fiber breakage during the dosing stage. However, to fully realize the potential of LFT composites, a proper design of the nozzle, molding channels, and the part is also essential.

## Figures and Tables

**Table 1:** Selected material properties from the manufacturer’s data sheet.

Glass Fiber Content (%)	Tensile Modulus (MPa)	Tensile Strength (MPa)	Elongation at Break (%)	Heat Deflection Temperature (°C)
50	10.500	115	1.9	158

**Table 2:** Studied combinations of injection molding parameters.

Limits	V_f_ [m/s]	P_back_ [bar]
Min	0.15	20
Max	0.4	50

**Table 3:** Fiber measurement results in relation to the combination of process parameters.

ID	P_back_ [bar]	V_f_ [m/s]	l_n_ [μm]	l_w_ [μm]	FLR
2	20	0.15	826	1480	1.79
4	20	0.4	903	1676	1.85
6	50	0.15	482	870	1.8
8	50	0.4	686	1265	1.84

## Data Availability

The data presented in this study are available on request from the corresponding author due to privacy policies.
